# Multi-Omics Insights into Regulatory Mechanisms Underlying Differential Deposition of Intramuscular and Abdominal Fat in Chickens

**DOI:** 10.3390/biom15010134

**Published:** 2025-01-15

**Authors:** Yuxin Xing, Chenglin Ma, Hongbo Guan, Jianing Shen, Ying Shen, Guoxi Li, Guirong Sun, Yadong Tian, Xiangtao Kang, Xiaojun Liu, Hong Li, Weihua Tian

**Affiliations:** 1 College of Animal Science and Technology, Henan Agricultural University, Zhengzhou 450046, China; xyx2022x@stu.henau.edu.cn (Y.X.); mcl2023@stu.henau.edu.cn (C.M.); ghb2023@stu.henau.edu.cn (H.G.); sjn2024@stu.henau.edu.cn (J.S.); sy2024@stu.henau.edu.cn (Y.S.); guoxili2023@henau.edu.cn (G.L.); grsun@stu.henau.edu.cn (G.S.); tianyadong@henau.edu.cn (Y.T.); xtkang2023@henau.edu.cn (X.K.); xiaojun.liu@henau.edu.cn (X.L.); 2 Key Laboratory of Livestock and Poultry Resources (Poultry) Evaluation and Utilization of Ministry of Agriculture and Rural Affairs, Henan Agricultural University, Zhengzhou 450046, China; 3 Henan Innovative Engineering Research Center of Poultry Germplasm Resource, Zhengzhou 450046, China

**Keywords:** intramuscular fat, abdominal fat, differential deposition, regulatory mechanisms, chicken

## Abstract

Excessive abdominal fat deposition in chickens disadvantages feed conversion, meat production, and reproductive performance. Intramuscular fat contributes to meat texture, tenderness, and flavor, serving as a vital indicator of overall meat quality. Therefore, a comprehensive analysis of the regulatory mechanisms governing differential deposition of abdominal versus intramuscular fat is essential in breeding higher-quality chickens with ideal fat distribution. This review systematically summarizes the regulatory mechanisms underlying intramuscular and abdominal fat traits at chromatin, genomic, transcriptional, post-transcriptional, translational, and epigenetic-modification scales. Additionally, we summarize the role of non-coding RNAs and protein-coding genes in governing intramuscular and abdominal fat deposition. These insights provide a valuable theoretical foundation for the genetic engineering of high-quality and high-yielding chicken breeds.

## 1. Introduction

Poultry is among the most widely consumed meats worldwide. However, intensive selection in broiler body weight and growth rate has lowered intramuscular fat (IMF, fat within/between muscle fibers and between muscle bundles) and increased abdominal fat percentage (AFP). These traits are not preferred by consumers and hinder profitable farming [1,2,3,4]. Because IMF directly influences meat color, flavor, juiciness, and tenderness, it serves as a pivotal indicator for evaluating meat quality [5,6]. In contrast, abdominal fat (AF) is treated as a waste of broiler processing. Its excessive accumulation diminishes feed conversion rate, growth performance, meat quality, and reproductive performance [7,8,9,10,11]. To better control fat deposition in a favorable manner and generate high-yield, high-quality broilers, the underlying regulatory mechanisms must be comprehensively analyzed.

With the continuous advancements in sequencing technology, in-depth exploration of the genetic and epigenetic factors regulating fat deposition has become feasible at multi-omics scales, such as genomics, transcriptomics, proteomics, and epigenetics, ranging from the molecular and cellular to the tissue and individual levels. Genomic research mainly involves the structure, variation, function and evolution of an organism’s entire genome [12]. Genomic selection technology is a powerful tool to achieve directed fat deposition in chicken breeding [13]. Transcriptomics technology mainly reveals differential gene expression across various stages or among diverse samples, and can identify hub genes that are closely related to fat deposition [14,15]. Proteomics technology can identify key proteins associated with fat deposition, which can serve as potential targets for the regulation of fat deposition and provide insights into their dynamics through post-translational modifications [16]. Epigenetic mechanisms can modulate gene expression without changes in DNA sequence and primarily encompass DNA methylation, RNA modifications (such as m^6^A methylation), three-dimensional chromatin interaction, chromatin accessibility, and non-coding RNAs [17,18]. Of these, DNA methylation can directly affect gene expression levels associated with fat deposition by regulating gene transcription and genome organization [19,20,21]. RNA methylation modifications impact RNA functions, including adipogenesis-related gene expression, stability, splicing, and translation efficiency [22,23]. Alterations in the three-dimensional chromatin structure and chromatin accessibility can affect the spatial proximity of genes to their distant regulatory elements, thereby regulating the expression levels of these genes [24,25]. Non-coding RNAs mainly consist of circular RNAs (circRNAs), long non-coding RNAs (lncRNAs), and microRNAs (miRNAs), and they play crucial regulatory roles in fat deposition by regulating specific adipogenesis-related gene expression. For example, miRNAs can partially or fully complementarily bind to the 3′ untranslated region (3′UTR) of mRNAs involved in fat synthesis, metabolism, and other related processes through their “seed region” (the 2–8 nucleotide sequence at the 5′ end of the miRNA) [26,27,28,29,30]. This binding can lead to the target mRNA degradation and translation inhibition, thereby affecting the process of fat deposition in the organism [26,27]. The purpose of this review paper is to systematically summarize the regulatory mechanisms and genetic/epigenetic factors underlying intramuscular and abdominal fat deposition at these omics scales, which provides a valuable theoretical foundation for the genetic breeding of high-quality and high-yielding chicken breeds.

## 2. Fat Deposition in Chickens

Adipocytes originate from the mesoderm and first differentiate into mesenchymal stem cells (MSCs) [31,32,33]. The simultaneous influence of multiple regulatory factors then triggers MSC transformation into preadipocytes [34,35]. Preadipocytes then differentiate and mature into adipocytes, guided by a cascade of transcriptional and endocrine factors [36]. Fat deposition primarily hinges on augmenting both the number and size of fat cells [37]. Adipocyte count is fixed by factors before birth and during early development. The subsequent fat deposition phase is primarily distinguished by lipid droplet formation, leading to an enlargement in adipocyte size [38].

Unlike mammals, over 90% of de novo fatty acid (FA) synthesis of chickens occurs in the liver [39]. First, hepatic FAs are converted into lipids including triglycerides (TGs) and cholesterol. These two compounds are then assembled by apolipoproteins into very low-density lipoproteins (VLDLs) and secreted into the blood [40]. After traveling through the peripheral vascular system to the abdomen, muscles, and subcutaneous tissue, VLDLs are hydrolyzed into FAs by lipoprotein lipase (LPL), re-absorbed by adipocytes and re-esterified to generate TG [41,42]. The aggregation of TG results in lipid droplet accumulation, eventually forming AF, IMF, and subcutaneous fat (SCAT) [43]. Deposition of IMF occurs first, followed by SCAT and AF. In terms of speed, AF deposition is the fastest, followed by SCAT and IMF [44]. In chickens, AF is more abundant than IMF because of its faster deposition rate and stronger lipid formation ability [45]. Additionally, AF has significantly more TG content in AF (349.7 mg/g) than in breast or leg muscle (12.3 mg/g and 24.8 mg/g) [46]. Preadipocytes derived from AF have a greater adipogenic differentiation ability than IMF-derived preadipocytes [47].

## 3. Relevance of IMF and AF

Several studies have demonstrated a significant positive correlation between IMF content and AFP in relation to phenotype and genetic coefficient. A study on 90-day-old purebred Beijing-You meat-type chicks (BJY) revealed positive correlations between IMF content and AFP phenotype, as well as between IMF content and body weight (BW) (rP = 0.11–0.330) [48]. Likewise, genetic parameters of IMF were highly correlated with those of BW (rA = 0.75) and of abdominal fat weight (AFW) (r = 0.66), as well as being moderately genetically correlated with AFP (r = 0.32) [48]. When broiler breeds with high and low AF content were bred, selecting those with high AF led to a corresponding rise in IMF content [49]. Moreover, a comparison of saturated and unsaturated fat diets (isocaloric and isonitrogenous) for broilers found that the former caused chickens to exhibit higher AFP and IMF content [50].

Notably, however, some data indicate a negative to no correlation between IMF and AFP phenotypes or genetic coefficients. Selection simulation with independent up-selection for IMF as a control demonstrated that balanced selection (with up-selection for IMF and down-selection for AFP) over five generations increased IMF by 11.4% and decreased AF by 1.5% among JXY chickens [51]. Another experiment on a fifth generation cohort selected for higher IMF showed that these chickens exhibited an 11.8% increase in IMF content, with no significant difference in AF rate from the randomly bred control line [52]. Another study on genetic parameters of fat deposition in white Plymouth rock hens and roosters revealed an estimated heritability of 0.71 for AFP, 0.24 for SCAT percentage, and 0.08 for IMF content. Additionally, the genetic correlation between AFW and IMF content was very low (0.02), while the genetic correlation between AFP and IMF content was negatively correlated (0.32) [53].

In addition to genetic factors, environmental variables such as diet also play an important role in fat deposition. Dietary supplementation of sea buckthorn fruit flavonoids [54] and L-carnitine [55] increases IMF content and lowers AF rate of broilers. Under heat stress, chromium supplementation reduces AFP without affecting IMF content [56]. All these findings taken together indicate that the relationship between IMF and AF is complex, with multiple genetic and environmental factors at play.

## 4. Differences Between IMF and AF

The two cell types differ in size, with IMF cells (diameter = 25–50 μm) being smaller than AF cells (diameter = 50–200 μm) [57,58]. Chicken abdominal preadipocytes possess a substantially higher capacity for adipogenic differentiation than chicken intramuscular preadipocytes [47,59,60]. This disparity means the deposition rate of AF is faster and more suitable as the primary energy storage site, enabling rapid responses to fluctuations in energy intake and expenditure. When compared with breast muscle, AF exhibits a notably higher TG content, alongside lower phospholipids (PLIP) and total cholesterol (TCHO) levels. In breast muscle, IMF consists predominantly of TG and PLIP, with lower TCHO content [7,60,61]. TG, as a crucial indicator of fat deposition, exhibits differing degrees of correlation with IMF (r = 0.45, *p* < 0.01) and AF (r = 0.65, *p* < 0.0001) [62,63,64]. Overall, IMF and AF exhibit remarkable differences in adipocyte size, lipid accumulation rate, and lipid composition.

## 5. Regulatory Mechanisms Underlying IMF Deposition

**Figure 1:**
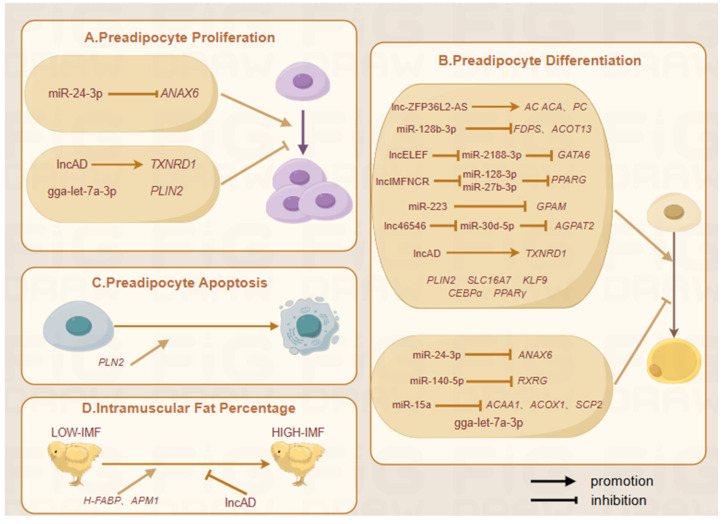
Genes and non-coding genes involved in IMF regulation. (**A**) Factors affecting preadipocyte proliferation; (**B**) Factors affecting preadipocyte differentiation; (**C**) Factors affecting preadipocyte apoptosis; (**D**) Factors affecting IMF percentage. miR-128b-3p, *ACOT13* [75]; *GPAM*, miR-223 [76]; *PLIN2* [77]; miR-128-3p, *FDPS* [78]; *SLC16A7* [79]; lncAD, *TXNRD1* [80]; *KLF9* [81]; *C/EBPα*, *PPARγ* [82]; *ApM1* [83]; miR-24-3p, *ANAX6* [84]; LncHLEF, miR-2188-3p, *GATA6* [85]; miR-140-5p, *RXRG* [86]; lncRNA-46546, miR-30d-5p, *AGPAT2* [87]; miR-15a, *ACAA1*, *ACOX1*, *SCP2* [88]; lncRNA IMFNCR, miR-128-3p, miR-27b-3p, *PPARG* [89]; lncRNA ZFP36L2-AS, *ACACA*, *PC* [90]; Gga-let-7a-3p [91].

Despite chicken IMF having low to moderate heritability (0.11–0.16) [51,65,66], artificial selection has considerably increased IMF content [51,52,67], highlighting the effectiveness of genetic approaches. To accelerate and improve breeding programs, we must therefore understand the genetic basis and regulatory mechanisms underlying IMF deposition in chickens, as well as develop molecular markers for selection. Omics analysis and histological techniques have identified candidate genes, non-coding RNAs, and signaling pathways responsible for IMF deposition. The main methods of investigation include genome-wide association analysis, transcriptome profiling, proteome analysis, single-cell RNA-seq, and epigenetic modification. Using chicken models of intramuscular preadipocyte proliferation and adipogenic differentiation, candidate genes and non-coding RNAs have been discovered that regulate intramuscular adipogenesis (Figure 1).

Zhang et al. (2023) conducted proteomic analyses on leg muscles of BYC at different developmental stages and identified *APOV1*, *VTG2*, and *VTG3* as crucial regulators for muscle lipid transport, and highlighted the importance of focal adhesion and ECM–receptor interactions in IMF deposition [68]. Cui et al. (2023) utilized whole-genome resequencing of 516 JingXing yellow chickens (JXY), single-cell RNA sequencing (scRNA-seq) of three 63-day old female JXY, and RNA-seq of breast muscle tissue of JXY chickens at various stages of development. The results show that *FSAN* is the key gene related to increasing IMF deposition. Additionally, de novo lipogenesis (DNL) plays an important role in IMF accumulation [67]. The use of scRNA-seq identified 10 cell clusters in the breast muscle of 5-day-old JYX and seven cell clusters in the breast muscle of 100-day-old JYX; the two developmental stages differed in aggregation patterns. Five myocyte-related clusters and two adipocyte clusters appeared on day 5, but only one myocyte-related cluster and one adipocyte cluster were observed on day 100. This pattern reflects a higher degree differentiation in end-stage cell boundaries. Up-regulated *APOA1* and *COL1A1* in adipocyte clusters were identified as chicken IMF cell biomarkers using RNA in situ hybridization [69]. Additionally, we employed high-through chromosome conformation capture (Hi-C) analysis to investigate the regulatory mechanism of muscle development and IMF deposition in chickens at a three-dimensional chromosomal level. By comparing the chromatin structure of chicken breast muscle from Chinese indigenous Lushi chicken (LS) and commercial Arbor Acres (AA) broiler chickens, we identified *IGF2BP3* and *HMGCR* regulated by topologically associating domain (TAD) boundary sliding as potential biomarkers for muscle development and IMF deposition [70].

Zhang et al. (2017) compared global DNA methylation profiles between juvenile hens (20 weeks old) and laying-period hens (55 weeks old) and revealed that older hens exhibit more IMF deposition and DNA methylation. The analysis identified 378 differentially methylated genes (DMGs) that are involved in muscle development, lipid metabolism, and aging. Hypermethylation and downregulation of *ABCA1*, *COL6A1*, and *GSTT1L* promoters in the older hens are probably responsible for significant differences in meat quality between the two age cohorts [71]. Zhang et al. (2020) isolated and cultured chicken intramuscular preadipocytes in vitro and constructed a model of intramuscular adipocyte differentiation, combined with DMGs identified using whole-genome sulfite sequencing (WGBS) and NA-SEQ techniques. The DMGs mainly affect fatty acid metabolism and muscle development; only *COL6A1* promoted IMF differentiation and inhibited cell proliferation [72]. Employing MeRIP-seq and RNA-seq techniques, Zhang et al. found that m^6^A peaks in breast and leg muscles of 180-day-old Jingyuan chickens were predominantly in the 3′ untranslated region (3′UTR) and coding sequence (CDS) regions; 176 DMGs were found between breast and leg muscles. These DMGs are significantly enriched in amino acids, peroxisomes, fatty acid biosynthesis, fatty acid extension, and cell adhesion molecule pathway biosynthesis; of particular note, *ECH1*, *BCAT1*, and *CYP1B1* are involved in regulating muscle lipid anabolic metabolism [73]. Yu et al. (2023) conducted MeRIP-seq and RNA-seq, comparing and analyzing transcriptome-wide m^6^A profiles and assessing IMF differences in breast muscles of Jingyuan chickens aged 42 days (group G), 126 days (group S), and 180 days (group M). Breast-muscle IMF content in the chest muscle increased significantly over time, while breast m^6^A peaks in the three groups were highly enriched in the CDS and 3′ UTR. In addition, they identified 129, 103, and 162 DMGs in breast muscle samples from the G, S, and M groups, respectively. These DMGs are involved in many physiological activities of muscle fat anabolism. Association analysis suggests that *LMOD2* is a regulator of differential IMF deposition [74].

## 6. Regulatory Mechanisms Underlying AF Deposition

**Figure 2:**
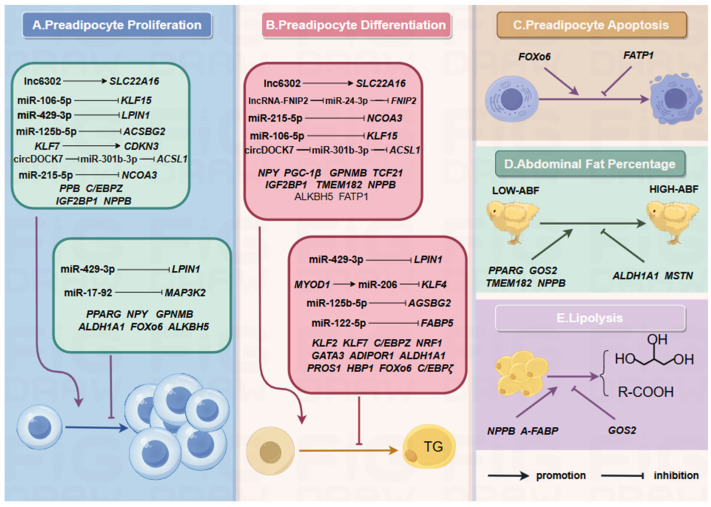
Genes and non-coding genes involved in AF regulation. (**A**) Factors affecting preadipocyte proliferation; (**B**) Factors affecting preadipocyte differentiation; (**C**) Factors affecting preadipocyte apoptosis; (**D**) Factors affecting AFP; (**E**) Factors affecting lipolysis. Lnc6302, *SLC22A16* [103]; *NPPB* [104]; *KLF7* [105,106], *CDKN3* [105]; *C/EBPZ* [107]; miR-106-5p, *KLF15* [108]; *IGF2BP1* [109]; miR-429-3p, *LPIN1* [110]; *ACSBG2*, miR-125b-5p [111]; *HBP1*, *SOCS3* [112]; *ACSL1*, circDOCK7, miR-301b-3p [113]; *FATP1* [114]; *NPY* [115]; *PGC-1β* [116]; *GPNMB* [117]; *TCF21* [118]; *KLF15*, miR-106-5p [108]; *TMEM182* [119]; *ALKBH5* [120]; *FNIP2*, LncRNA-FNIP2, miR-24-3p [121]; *ALDH1A1* [122]; *FoxO6* [123]; miR-17-92, *MAP3K2* [124]; *KLF2* [125]; *NRF1* [126]; *GATA3*, *KLF7* [127]; *ADIPOR1* [128]; *PROS1* [129]; *MYOD1*, miRNA-206, *KLF4* [130]; *C/EBPζ* [131]; *FABP5*, miR-122-5p [132]; *G0S2* [133]; *MSTN* [134]; *A-FABP* [135]; *HBP1* [136].

AFW and AFP are direct indicators for assessing AF deposition. In addition to being positively correlated, their heritability is high (0.62 and 0.24, respectively [48]). The high heritability and strong positive genetic correlation between AFW and AFP indicate that genetic selection is the primary approach to alleviate excessive abdominal fat deposition in chickens. Recent advancements have identified a series of candidate genes, non-coding RNAs, and regulatory factors associated with abdominal fat deposition in chickens using multi-omics techniques, including genomics, transcriptomics, proteomics, single-cell transcriptomics, and epigenetics. Through the use of chicken abdominal preadipocyte proliferation and adipogenic differentiation models, some genes and non-coding RNAs are investigated for their influence on abdominal fat deposition (Figure 2).

Wang et al. (2021) [92] identified 199 differentially abundant proteins (DAPs) and 2167 differentially abundant genes (DEGs) through integrated RNA-seq, and quantitative proteome analyses of AF from chickens with high and low AF levels. Among them, down-regulated DEGs and DAPs were mainly enriched in pathways related to fatty acid metabolism, fatty acid biosynthesis, and PPAR signaling, whereas up-regulated DEGs and DAPs enriched the lysosome pathway. In addition, comparing the genes that overlapped across DEGs and DAPs identified 32 key genes (*MAP4*, *ABHD12B*, *ACACA*, etc.) that regulate AF deposition [92]. Wu et al. (2021) [93] identified 22 DEPs through proteomic analysis of hepatic tissues from chickens with high and low AF levels at different ages; of these, seven DEPs were highly expressed in high-AF chickens and 15 DEPs were highly expressed in low-AF chickens. FABP1 and ECSH1 are involved in lipid metabolism. High FABP1 expression is associated with increased hepatic VLDL synthesis and abdominal lipid deposition, whereas low expression is linked to inhibited fatty acid oxidation and enhanced abdominal lipid deposition [93]. Li et al. (2021) [94] used scRNA-seq to analyze 25,071 transcriptomes of AF-derived preadipocytes from chicks raised on melatonin-added drinking water. They then classified cells into subpopulations and defined them as 14 clusters using tSNE analysis. The G0S2+ cluster and the G0S2− cluster were the largest components of normal and melatonin-treated preadipocytes, respectively; the clusters were significantly upregulated in the former and downregulated in the latter. Additionally, melatonin promotes AF decomposition by downregulating *G0S2* expression, while upregulating *FABP4* expression inhibits AF synthesis [94].

Sun et al. (2014) [95] compared the *PPARγ* promoter methylation levels and gene expression in Northeast Agricultural University broiler lines divergently selected for abdominal fat content (NEAUHLF) at 2, 3, and 7 weeks of age. They revealed differential methylation at three CpG sites (−548, −435, and −383 bp) of *PPARγ* between lean (low AFP) and fat (high AFP) broiler lines. Additionally, methylation of the *PPARγ* promoter decreased as chickens aged. Notably, *PPARγ* expression was significantly lower in lean broilers than in fat broilers across all three time periods, and its expression was negatively correlated with DNA methylation (r = −0.653). In summary, DNA methylation influences regulation of chicken *PPARγ* during adipose tissue development [95]. Gao et al.’s (2015) [96] study showed that the methylation percentage of the *CEBPA* promoter was significantly higher in lean broilers than in fat broilers (chicken is from NEAUHLF) at 2, 3 and 7 weeks of age. At 2 weeks, methylation percentage was negatively correlated with *CEBPA* expression (r = −0.8312). The results suggest that *CEBPA* methylation in adipose tissue may regulate early fat development in chickens [96]. Similarly, Sun et al.’s (2019) [97] study based on NEAUHLF showed that *PLIN1* expression in adipose tissue of obese broilers aged 1 to 7 weeks was significantly higher than that of lean broilers, and was significantly positively correlated with chicken AFP (r = 0.627). At 5 and 6 weeks, the CpG5 DNA methylation level of lean broilers only at the −490 bp position was significantly higher than that of fat broilers, and was negatively correlated with *PLIN1* mRNA and AFP levels [97]. Wu et al. (2019) [98] showed that *APOA1* was differentially expressed in abdominal adipose tissues of lean and fat broilers. Additionally, methylation of *APOA1* promoter CpG islands was negatively correlated with *APOA1* mRNA expression. In vitro methylation of the *APOA1* promoter containing CpG islands and CpG methyltransferase leads to transcriptional inhibition. Therefore, demethylation of the *APOA1* promoter CpG island enhances *NRF1* activation of *APOA1* transcription [98]. Zhang et al. (2020) [99] analyzed *KLF7* DNA methylation and its relationship with AF transcripts and blood metabolic indices of 21 one-day-old male Chinese fast-growing yellow broilers. The results revealed that *KLF7* transcripts were negatively correlated with blood glucose levels (r = −0.61841). Furthermore, DNA methylation of the promoter region was notably lower than that of exon 2. Correlation analysis showed that the methylation of PCpG6 and E2CpG9 at 26 CpG sites in promoter and exon 2 was significantly correlated with *KLF7* transcript and blood HDL levels, respectively. Therefore, DNA methylation and gene expression exhibit a complex interplay in chicken AF [99].

Cheng et al. (2021) [100] used MeRIP-seq to map and compare the m^6^A modification landscape of AF tissue from chickens with high and low AFP. The modification appeared with high frequency in start codons, stop codons, coding regions, and 3′-untranslated regions. Highly m^6^A-methylated genes (high AFW chickens vs. low AFW chickens) were primarily associated with fatty acid biosynthesis and fatty acid metabolism, while lowly m^6^A-methylated genes were mainly involved in developmental processes [100]. Zhou et al. (2022) [101] compared 30-day-old male Xueshan chickens injected with CORT (2 mg/kg twice daily for 14 days) and without CORT. The treatment increased plasma triglyceride concentration, and AF cell diameter. Global m^6^A methylation significantly decreased in CORT-treated chickens. Notably, significant differences in site-specific m^6^A demethylation were observed in exon7 of *PPARA* mRNA, and a mutation of the m^6^A site in the *PPARA* gene fused with GFP revealed that demethylated RRACH in *PPARA* CDS impaired protein translation in vitro. These results indicated that m^6^A-mediated *PPARA* translational suppression contributes to CORT-induced AF deposition in chickens [101]. Chao et al. (2024) [102] divided six 100-day-old chicks into high and low AFP groups, performed MeRIP-seq and RNA-seq on their AF, and identified 16 DEGs associated with m^6^A modifications. *ELOVL2*, *PDK4*, *PMP2*, *FABP1*, *LAMP3*, *LCAT*, and *SLC2A1* are associated with adipogenesis. *LCAT* was down-regulated concurrently with decreased mRNA methylation in the low-fat group; this gene inhibits preadipocyte proliferation and promotes preadipocyte differentiation. Moreover, *ALKBH5* mediates RNA stability of *LCAT* through demethylation and thereby affects adipogenesis in chickens [102].

## 7. Regulatory Mechanisms Underlying Differential Deposition of IMF and AF

Multi-omics techniques have been successfully applied to identify genes and signaling pathways responsible for differential IMF and AF deposition in chickens. Zhang et al. (2019, 2020) conducted RNA-seq on chicken AF-derived preadipocytes and IMF-derived preadipocytes before and after differentiation. Their results revealed significant differences in the expression patterns of circular RNA, long non-coding lncRNA, microRNA, and mRNA between the two types of preadipocytes. Moreover, DEGs during IMF-derived preadipocyte differentiation were significantly enriched in pathways such as PPAR and ECM receptor interaction, while DEGs during AF-derived preadipocyte differentiation were significantly enriched in pathways such as steroid biosynthesis pathway, calcium signaling pathway, and ECM receptor interaction [47,80,137]. With RNA-seq, Ma et al. (2021) analyzed differences in gene expression patterns between chicken AF-derived preadipocytes and IMF-derived preadipocytes. The findings indicated that *APOA1*, *ADIPOQ*, *FABP3*, *FABP4*, *FABP7*, *HMGCS2*, and *LPL* expressions in IMF-derived preadipocytes were significantly lower than those in AF-derived preadipocytes. This pattern may explain why IMF-derived preadipocytes have a lower adipogenic differentiation ability than AF-derived preadipocytes [60]. Liu et al. (2018) [46] found that TG content was significantly higher in AF tissue of 90-day-old BYC than in muscle tissues. Relatedly, the expressions of fat metabolism-related genes *PPARG*, *PPARGC1B*, *FABP1*, *FABP4*, *LPL*, *FASN*, *DGAT2*, and *ACSL1* in AF tissue were significantly higher than in muscle tissues [46]. Luo et al. (2022) [138] used RNA-seq and WGCNA to demonstrate that genes related to pyruvate and citric acid metabolism (e.g., *LDHA*, *GPX1* and *GBE1*) were mainly involved in IMF deposition in Wenchang chicken breast muscle. Genes related to acetyl-CoA and glycerol metabolism (e.g., *FABP1*, *ELOVL6*, *SCD*, and *ADIPOQ*) were mainly involved in AF deposition. In addition, carbohydrate metabolism signaling plays an important regulatory role in the IMF deposition, while fatty acid metabolism and glycerol metabolism signaling pathways are critical in regulating AF deposition [138]. Another study using RNA-seq and WGCNA found that *L3MBTL1*, *TNIP1*, *HAT1*, and *BEND6* expressions are associated with high IMF content and low AFW. The study also identified *ACSM3* and *CYP2AB1* as candidate genes for increasing IMF content and decreasing AFW in chicken breast muscle [139]. Li et al. (2023) [140] performed RNA-seq and lipidomic analysis of breast muscle and AF tissues from three chicken breeds and identified 4737 DEGs. These DEGs, which are involved in glycerophospholipid metabolism and glycerolipid metabolism, were associated with differences in lipid metabolite accumulation between IMF and AF. Additionally, the most important pathway involved in tissue-specific lipid deposition was PPAR signaling [140]. Previously, we conducted RNA-seq and non-targeted lipomic analysis on breast muscle and AF tissues, detecting 423 differential lipid molecules (DLMs) between breast muscle and AF. Further integration of RNA-seq data with WGCNA revealed 386 unique genes implicated in promoting IMF deposition in breast muscle, 213 unique genes implicated in promoting AF deposition, and 6 unique genes implicated in inhibiting AF deposition. In addition, seven genes are related to the promotion of IMF deposition and the inhibition of AF deposition, while 28 genes are related to the promotion of IMF deposition but do not affect AF deposition [141].

Because the liver is the main site of lipid metabolism in chickens, Xing et al. (2021) [142] conducted WGCNA on RNA-seq data of livers from JXY chicken at different developmental stages. *MFGE8*, *HHLA1*, *CKAP2*, and *ACSBG2* were the main genes that demonstrated a positive correlation with AFW [142]. Moreover, transcriptome and proteome profiling on fat and lean chicken livers at five embryonic stages revealed variations in mRNA and proteins associated with lipid transport (FABP2, NDK, APOA1), lipid scavenging (HSPB1), and energy metabolism (NDUFB10). These variations likely account for post-birth differences in AF deposition [143]. RNA-seq and proteome sequencing have identified numerous candidate genes regulating individual variation in fat deposition of liver, breast muscle, and AF tissue. Such techniques have also been applied to determine candidates influencing AF-derived preadipocytes and IMF-derived preadipocytes at different stages of differentiation. However, all of these studies mainly focus on a single tissue or single cell type. Little to no data exist regarding inter-tissue communication or intercellular communication during fat deposition. Notably, our previous study found that hepatocyte exosomes delivered lncRNA-lncHLEF to chicken IMF-derived preadipocytes and AF-derived preadipocytes. This process promotes adipogenic differentiation and deposition of IMF-derived preadipocytes, but not AF-derived preadipocytes [85]. Hence, inter-tissue communication likely regulates differential IMF and AF deposition. These findings suggest that IMF and AF deposition in chickens is governed by distinct mechanisms, providing a foundation for breeding broiler chickens with high IMF and low AF.

## 8. Conclusions and Outlook

In conclusion, both IMF and AF are complex traits under the control of multiple genetic/epigenetic factors. They differ significantly in deposition rate and regulation mechanisms. Candidates including genes, non-coding RNAs, chromatin remodeling, and epigenetic modification have been identified to participate in the differential deposition of IMF and AF, providing a theoretical basis for genetic breeding of chickens. However, very few studies have investigated the regulatory roles and molecular mechanisms of these important candidates underlying the differential deposition of IMF and AF in vitro, resulting in the limitation of their effective application in chicken genetic breeding. Additionally, existing studies tended to focus on the biological functions of single genes in IMF and AF deposition, neglecting the fact that fat distribution throughout the body is regulated by multiple coordinated factors. In particular, tissue–tissue crosstalk plays an indispensable role in within-tissue pathways. To the best of our knowledge, no studies to date have explored inter-tissue communication during the regulation of AF and IMF deposition in chickens. Therefore, to clarify the molecular regulatory mechanisms governing differential deposition of IMF and AF in chickens, systematic biology should also be used to uncover more candidate genes and relevant pathways and investigate their biological functions and regulatory mechanisms. This will help identify functional biomarkers for the genetic breeding of high-efficient and high-quality chicken breeds.

## Data Availability

No new data were created or analyzed in this study.
